# NANOG expression correlates with differentiation, metastasis and resistance to preoperative adjuvant therapy in oral squamous cell carcinoma

**DOI:** 10.3892/ol.2013.1690

**Published:** 2013-11-19

**Authors:** MASAHIRO WATANABE, YUICHI OHNISHI, HIROSHI INOUE, MASAHIRO WATO, AKIO TANAKA, KENJI KAKUDO, MASAMI NOZAKI

**Affiliations:** 1 Graduate School of Dentistry, Osaka Dental University, Hirakata, Osaka 573-1121, Japan; 2 Department of Cell Biology, Research Institute for Microbial Diseases, Osaka University, Suita, Osaka 565-0871, Japan; 3 Second Department of Oral and Maxillofacial Surgery, Osaka Dental University, Hirakata, Osaka 573-1121, Japan; 4 Department of Dentistry and Maxillofacial Surgery, Osaka Red Cross Hospital, Osaka 543-8555, Japan; 5 Oral Pathology, Osaka Dental University, Hirakata, Osaka 573-1121, Japan

**Keywords:** oral squamous cell carcinoma, NANOG, differentiation, metastasis, preoperative adjuvant therapy

## Abstract

NANOG protein, a transcription factor expressed in embryonic stem cells, is overexpressed in tumor development. Although studies investigating the function of NANOG in cancer have shown that it plays several roles, such as in cell proliferation, invasion and metastasis, the overall function of NANOG in cancer cells has remained elusive. In the present study, NANOG expression in oral squamous cell carcinoma (OSCC) was examined to determine its potential clinical significance. The expression of NANOG protein was assessed in 60 patients with OSCC by immunohistochemistry, and its correlation with clinicopathological factors and metastasis was evaluated. NANOG protein levels in human OSCC cell lines were determined by western blotting and immunofluorescence staining. NANOG protein expression was identified in 52 cases (86.7%) and expression levels were higher in primary foci of poorly differentiated OSCC than in those of well-differentiated OSCC, indicating that NANOG expression is associated with OSCC differentiation. Regardless of the differentiation levels of primary foci, NANOG expression levels in metastatic foci were extremely high. In addition, NANOG expression in metastatic foci was maintained at high levels following preoperative adjuvant therapy. Furthermore, NANOG protein was detected at an identical level in human OSCC cell lines. These data indicate that NANOG-expressing OSCC cells tend to metastasize and that metastatic tumors expressing NANOG may be resistant to preoperative adjuvant therapy, including chemoradiation. Thus, assessment of NANOG expression may assist the strategy for treatment of OSCC metastasis.

## Introduction

Head and neck squamous cell carcinoma, which includes oral squamous cell carcinoma (OSCC), is the sixth most prevalent malignancy worldwide (1,2). Due to the poor prognosis of OSCC, the overall five-year survival rate of patients following surgical resection has not improved markedly during the past three decades (3).

The transcription factor NANOG is critical for the regulation of cell fate in the inner cell mass during embryonic development and pluripotency of embryonic stem cells (4–7). Overexpression of NANOG protein has been previously found in a variety of tumors, including breast cancer (8), colorectal cancer (9,10), gastric carcinoma (11) and OSCC (12,13). Previous studies report variable NANOG expression, from undetectable to extremely high levels, in OSCC samples. Furthermore, NANOG expression may be associated with patient survival. Elevated NANOG expression has been found to be associated with a poor prognosis, advanced stage and medially-to-poorly differentiated OSCC (14,15). Based on these observations, NANOG may be a useful prognosis factor. However, the correlation among NANOG expression, differentiation and metastasis in OSCC remains unclear.

In this study, NANOG expression in OSCC specimens was examined by immunohistochemistry. Furthermore, the association between NANOG expression and differentiation, metastatic potency and resistance of OSCC to preoperative adjuvant therapy was evaluated.

## Materials and methods

### Patients

Between 1997 and 2011, 60 patients with operable oral cancer underwent surgery at the Department of Oral and Maxillofacial Surgery (Osaka Dental University Hospital Hirakata, Japan; Table I). This study follows the tenets of the Declaration of Helsinki and was approved by the ethics committee of Osaka Dental University Hospital (Osaka, Japan). Informed consent was obtained from the patients. None of the primary foci were subjected to preoperative adjuvant therapy and, among 24 metastatic samples, 11 were from patients who underwent preoperative adjuvant therapy. The constituents of the adjuvant therapy are shown in Table II. Tumors were evaluated histologically, based on the International Union Against Cancer classification (16).

### Immunohistochemistry

Tissue samples of oral cancers of various stages from patients were fixed in 10% neutral-buffered formalin solution immediately following resection and were embedded in paraffin. Sections of 4-μm thickness were cut and mounted on silane-coated glass slides. The sections were deparaffinized in d-limonene and dehydrated in a graded ethanol series. Antigen retrieval was performed by autoclaving at 121°C for 15 min in Tris-EDTA buffer (pH 9.0). Endogenous peroxidase activity was blocked by incubation with 3% H_2_O_2_ for 10 min and nonspecific reactions were blocked by incubation with blocking solution (Nacalai Tesque, Inc., Kyoto, Japan) for 10 min. The tissue sections were incubated with goat anti-NANOG polyclonal antibody (1:300; Abnova, Taipei, Taiwan) at room temperature for 1 h. Tissue sections were then incubated with anti-goat IgG peroxidase-conjugated micropolymer (Vector Laboratories, Burlingame, CA, USA) at room temperature for 30 min and visualized by incubation with 3,3′-diaminobenzidine tetrahydrochroride liquid system (Dako, Tokyo, Japan) at room temperature for 5 min. The sections were counterstained with hematoxylin and observed by light microscopy (Olympus Corporation, Tokyo, Japan).

### Evaluation of slides

NANOG protein immunoreactivity was evaluated by two independent pathologists who had no knowledge of the patient’s clinicopathological factors and outcomes. Nuclear expression of NANOG protein was scored semiquantitatively by the combination of intensity (1, weak staining; 2, moderate staining; and 3, strong staining) and the proportion of positively stained tumor cells per 1,000 tumor cells in high-power fields (1, <25%; 2, 25–50%; 3, 51–75%; and 4, >75%). The sum of the staining intensity and percentage of positive tumor cell scores was graded as follows: +, 2–3; ++, 4–5; and +++, 6–7. There were no discrepancies between the two pathologists in the overall interpretation of the immunohistochemistry results.

### Statistical analysis

Mann-Whitney U tests were performed using the SPSS software (version 13.0; SPSS, Inc., Chicago, IL, USA) to identify statistically significant differences between samples. Data are presented as the mean ± SD. P<0.05 was considered to indicate a statistically significant difference.

### Cell culture

Human SAS, HSC-3 and HSC-4 OSCC cell lines (RIKEN BioResource Center, Ibaraki, Japan) were cultured in DMEM supplemented with 10% fetal calf serum (both Invitrogen Life Technologies, Carlsbad, CA, USA) at 37°C in a humidified atmosphere of 95% air and 5% CO_2_. Cell monolayers were prepared by plating on 10-cm cell culture dishes (Asahi Glass, Tokyo, Japan).

### Western blotting

Proteins were resolved in RIPA Buffer [150 mM NaCl, 1.0% Triton X-100, 0.5% sodium deoxycholate, 0.1% SDS and 50 mM Tris-HCl (pH 8.0)] and separated by 10% SDS-PAGE. A rabbit anti-NANOG antibody (Abcam, Cambridge, UK) was used as the primary antibody and peroxidase-linked ECL anti-rabbit IgG (GE Healthcare Japan, Tokyo, Japan) was used as the secondary antibody. ECL plus (GE Healthcare Japan) was used as the substrate for western blotting.

### Immunofluorescence staining

Cultured cells were fixed with 3.5% formaldehyde, permeabilized with 0.2% Triton X-100 and blocked with Image-iT™ FX Signal Enhancer (Invitrogen Life Technologies). Rabbit anti-NANOG antibody (Abcam) was used as the primary antibody. Next, Alexa Fluor 594-conjugated IgG (Molecular Probes, Eugene, OR, USA) was used as the secondary antibody. Following incubation with the antibodies, SlowFade^®^ Gold antifade reagent with 4′,6-diamidino-2-phenylindole (Invitrogen Life Technologies) was added and coverslips were mounted. The specimens were observed using a laser scanning confocal microscope (FV10i-DOC; Olympus Corporation).

## Results

### NANOG protein expression in OSCC patients and OSCC cell lines

**Figure 1:**
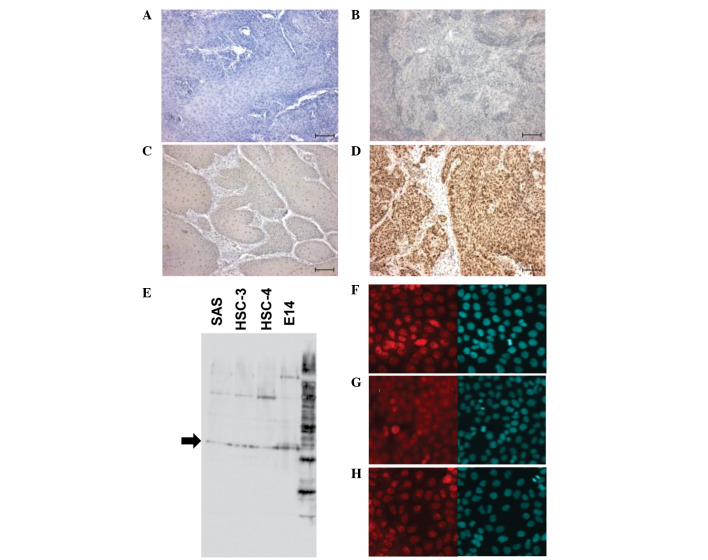
Expression of NANOG protein in OSCC tissues and cell lines. (A) Negative expression of NANOG in OSCC tissues (−). (B) Weak expression of NANOG in OSCC tissues (+). (C) Moderate expression of NANOG in OSCC tissues (++). (D) Strong expression of NANOG in OSCC tissues (+++) (scale bars, 100 μm). (E) Western blot analysis of NANOG protein expression in SAS, HSC-3 and HSC-4 cells. Nanog protein in mouse embryonic stem cells (E14) was used as a positive control for western blotting (arrow indicates 35 kDa). Immunocytochemical analysis of NANOG protein expression in (F) SAS, (G) HSC-3 and (H) HSC-4 cells. Right panels show 4′,6-diamidino-2-phenylindole staining in the nuclei of the cells. OSCC, oral squamous cell carcinoma.

NANOG protein was clearly stained in the nuclei of cells at various levels in OSCC specimens. Among 60 paraffin-embedded OSCC tissues of primary focus, eight cases (13.3%) were negative (−), 15 (25%) showed weak expression (+), 22 (36.7%) showed moderate expression (++) and 15 (25%) showed strong expression (+++). Representative cases of the different NANOG protein expression levels are shown in Fig. 1A–D. To confirm the expression of NANOG in OSCC cell lines, NANOG protein levels were analyzed in SAS, HSC-3 and HSC-4 cells derived from tongue SCCs by western blotting and immunofluorescence staining. NANOG protein was detectable at the same levels in all three cell lines (Fig. 1E–H).

### High NANOG protein expression in poorly differentiated OSCC and metastatic foci of OSCC

**Figure 2:**
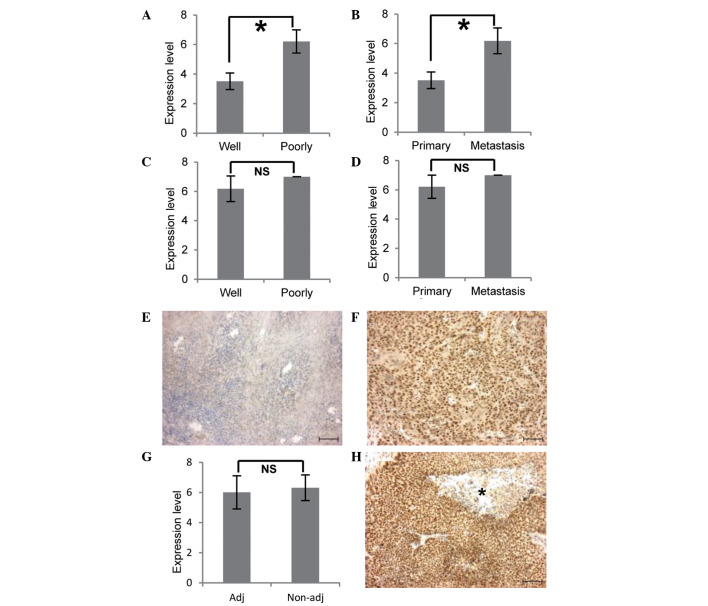
High level NANOG expression in tissue samples from metastatic foci in the OSCC patients. (A) Difference in NANOG expression levels between well-differentiated and poorly differentiated primary foci. (B) Difference in NANOG expression levels between primary and metastatic foci of well-differentiated OSCC. (C) Difference in NANOG expression levels between well-differentiated and poorly differentiated metastatic foci. (D) Difference in NANOG expression levels between primary and metastatic foci of poorly differentiated OSCC. (E) Weak (+) NANOG expression in primary focus. (F) Strong (+++) NANOG expression in the metastatic focus. (G) Difference in NANOG expression levels in metastatic foci between patients who received preoperative adjuvant therapy (Adj) and those who did not (Non-adj). (H) NANOG overexpression in a metastatic focus of a patient who received preoperative adjuvant therapy. Asterisk indicates necrotic tissue. Where applicable, data are presented as the mean ± SD (^*^P<0.01; Mann-Whitney U test) and scale bars represent 100 μm. OSCC, oral squamous cell carcinoma; NS, not significant.

NANOG protein expression levels were higher in primary foci of poorly differentiated OSCC than in those of well-differentiated OSCC (P<0.01; Table III; Fig. 2A). However, NANOG expression did not correlate with gender, region or T (stage of primary tumor) and N (stage of lymph node metastasis) status (P>0.05; Table III). In well-differentiated OSCC, NANOG expression in metastatic foci was elevated in comparison with its level in primary foci (P<0.01; Table III; Fig. 2B). Representative cases are shown in Fig. 2E and F. However, in metastatic foci, there was no significant association between NANOG expression and differentiation levels (P>0.05; Table III; Fig. 2C). Similarly, among primary and metastatic foci of poorly differentiated OSCC, no significant differences according to NANOG expression were identified (P>0.05; Table III; Fig. 2D).

### High NANOG expression is maintained in metastatic foci with preoperative adjuvant therapy

To investigate the association between NANOG expression and preoperative adjuvant therapy in OSCC, NANOG levels in metastatic lymph nodes were compared between patients who received preoperative adjuvant therapy and those who did not. There was no significant difference between the two groups (P>0.05; Table III; Fig. 2G). Moreover, OSCC cells (except for those in necrotic tissue) in metastatic lymph nodes subjected to adjuvant therapy expressed NANOG at high levels (Fig. 2H).

## Discussion

Our results show that the nuclei of cancer cells in the majority of OSCC samples (86.7%) were NANOG-positive. NANOG protein expression levels were higher in poorly differentiated OSCC than in well-differentiated OSCC, and NANOG was detected in all nuclei of OSCC cell lines examined. Furthermore, regardless of preoperative adjuvant therapy, NANOG expression in metastatic foci was extremely high. Although a number of the primary foci (13.3%) were negative for NANOG expression, all corresponding metastatic foci expressed high levels of NANOG.

Previous studies have shown that almost all tumors are heterogeneous (17–21). Cancer stem cells (CSCs), a small subpopulation of tumor cells, are the main factor in the initiation, growth, metastasis (14) and resistance of chemotherapy of tumors (22), therefore, cancer tends to recur. NANOG protein has been reported to be important in various tumor types, including OSCC (50% of primary foci and 66.7% of metastatic foci express NANOG protein) (14), colorectal cancer (20% of tumors) (10) and gastric carcinoma (10% of tumors) (11). In the present study, overexpression of NANOG was detected in poorly differentiated OSCC. Well-differentiated OSCC consists of numerous differentiated cells in the central region of the tumor and the majority undifferentiated tumor cells expressing NANOG exist at the fringe of focus. Immunofluorescence staining showed that OSCC cell lines expressed NANOG protein at the same level. These data indicate that NANOG is expressed not only in CSCs, but also in a large proportion of OSCC cells that are undifferentiated and highly proliferative. Previous studies indicate that NANOG promotes dedifferentiation of p53-deficient mouse astrocytes into brain cancer stem-like cells (23) and blocks differentiation, indicating that, in addition to its importance in CSCs, NANOG plays a significant role in maintaining the non-differentiation or proliferation of OSCC cells. Although the sensitivity of the present immunohistochemistry technique was higher than that in previous studies, specific samples were negative and all were confirmed to express the cell cycle marker Ki-67 (data not shown). These data indicate that NANOG is associated with proliferation, independently of the cell cycle, in undifferentiated OSCC cells, including CSCs. In OSCC patients with primary foci in which there was no expression of NANOG, metastatic foci markedly expressed NANOG. As aforementioned, NANOG-negative tumors may contain a limited number of undifferentiated OSCC cells, including CSCs. A previous study showed that high expression of NANOG was associated with metastasis (14). Therefore, in NANOG-negative patients, CSCs expressing NANOG in early stage primary foci metastasize and form the secondary tumor. Thereafter, NANOG-positive undifferentiated cancer cells may be maintained in metastatic foci and disappear from primary foci.

Sentinel lymph node biopsy, which is commonly used to aid breast cancer and melanoma staging, is effective in the diagnosis of OSCC metastasis (24). Immunohistochemistry is required to identify micrometastases and isolated tumor cells (25). The present study indicates that assessment of NANOG protein levels may be useful in sentinel lymph node biopsy.

In the present study, metastatic foci, with or without preoperative adjuvant therapy, showed extremely high expression of NANOG, although necrotic tissues were present within tumors in metastatic lymph nodes subjected to adjuvant therapy. These data indicate that specific tumor cells were necrotized by preoperative adjuvant therapy and that surviving NANOG-positive tumor cells proliferated. A previous study showed that preoperative adjuvant therapy for oral cancer did not significantly improve the survival rate despite the primary local control rate being improved (26). NANOG expression is positively associated with chemoresistance of OSCC (12,13), and CSCs express high levels of NANOG and exhibit high levels of chemoresistance (22). Thus, it is possible that specific tumor cells that did not express NANOG underwent cell death, while undifferentiated tumor cells, including CSCs overexpressing NANOG, survived and continued to proliferate in patients who underwent preoperative adjuvant therapy.

The results of the present study demonstrate that undifferentiated cancer cells overexpressing NANOG are important for metastatic OSCC. Therefore, we hypothesize that targeting NANOG protein may be a useful strategy for the treatment of OSCC metastasis.

## Figures and Tables

**Table I:** Clinicopathological factors in 60 patients with OSCC.

Variable	Well-differentiated	Poorly differentiated
Gender, n		
Male	18	18
Female	19	5
Age, years		
Mean	65.6	63.5
Range	18–84	47–81
Region, n		
Tongue	20	5
Gingiva	10	11
Floor of oral cavity	2	6
Buccal mucosa	4	1
Palate	1	0
T status, n		
T1	11	5
T2	17	12
T3	8	3
T4	1	3
N status, n		
N0	20	16
N1	6	1
N2a	0	0
N2b	11	6
N3	0	0

OSCC, oral squamous cell carcinoma.

**Table II:** Preoperative adjuvant therapy regimen.

Patient no.	Differentiation level	Regimen
1	Well-differentiated	PEP + RT
2	Well-differentiated	PEP + CDDP + TS-1 + RT
3	Well-differentiated	TS-1 + RT
4	Poorly differentiated	PEP + RT
5	Poorly differentiated	CDDP + 5-FU
6	Poorly differentiated	TS-1 + RT
7	Poorly differentiated	PEP + RT
8	Poorly differentiated	CDDP + 5-FU + RT
9	Well-differentiated	PEP + RT
10	Well-differentiated	PEP + CDDP + RT
11	Well-differentiated	PEP + CDDP + RT

PEP, pepleomycin; RT, radiation therapy; CDDP, cisplatin; TS-1, tegafur + gimeracil + oteracil potassium; 5-FU, 5-fluorouracil.

**Table III:** Correlation between NANOG expression and clinicopathological factors in 60 patients with OSCC.

			Expression, n			
				
Variable	Negative, n	Positive, n	+	++	+++	P-value
Total patients	8	52	15	22	15	
Gender						
Male	4	32	10	8	14	NS
Female	4	20	5	14	1	
Region						
Tongue	0	25	9	12	4	NS
Gingiva	5	16	4	6	6	
Floor of oral cavity	1	7	1	1	5	
Buccal mucosa	2	3	0	3	0	
Palate	0	1	1	0	0	
T status						
T1	1	15	3	9	3	NS
T2	4	25	9	9	7	
T3	1	10	3	4	3	
T4	2	2	0	0	2	
N status						
N1	0	5	0	2	3	NS
N2a	0	0	0	0	0	
N2b	0	8	0	1	7	
N3	0	0	0	0	0	
Primary tumor						
Well-differentiated	4	33	15	18	0	P<0.01
Poorly differentiated	4	19	0	4	15	
Metastasis						
Well-differentiated	0	11	0	3	8	NS
Poorly differentiated	0	2	0	0	2	
Recieved adjuvant therapy	0	11	0	4	7	NS
No adjuvent therapy	0	13	0	3	10	

OSCC, oral squamous cell carcinoma; T, stage of primary tumor; N, stage of lymph node metastasis; +, weak; ++, moderate; +++, strong.
